# Trapezoid fracture caused by assault

**DOI:** 10.4103/0019-5413.32053

**Published:** 2007

**Authors:** VA Malshikare, AV Oswal

**Affiliations:** Department of Orthopedics and Trauma, King Edward Memorial Hospital, Rasta Peth, Pune, India

**Keywords:** Assault, fracture trapezoid bone, internal fixation

## Abstract

In this report we describe an open fracture of trapezoid and break in anterior cortex of capitate due to assault in a young adult male. Direct impact force of a sharp object to the first web space caused the above fractures. Open reduction and internal fixation of the trapezoid was carried out using Kirschner wires. Cut extensor tendons, extensor retaniculum, capsule, adductor pollicis muscle, first dorsal interosseous muscle, soft tissue and overlying skin were sutured primarily. Three months after the operation the patient has made a complete recovery. There is no similar case reported in the literature.

## INTRODUCTION

Isolated trapezoid fracture is extremely rare. We hereby present a case of trapezoid fracture in view of its rarity.

## CASE REPORT

A 22-year-old man was assaulted by a sharp object. While attempting to protect himself he sustained an injury to the right hand. He presented with a sharp clean lacerated wound and swelling on first web space, which was extending around the right thumb from the proximal palmar crease to the distal carpal row dorsally with an open fracture of the trapezoid.

There was near full thickness injury to the adductor pollicis muscle, first dorsal interosseous muscle, capsule of the second carpometacarpal joint and extensor retinaculum. The tendons of extensor carpi radialis brevis (ECRB), extensor indices and extensor digitorum of the second and third fingers were also ruptured. However, there were no neurovascular complications.

**Figure 1:**
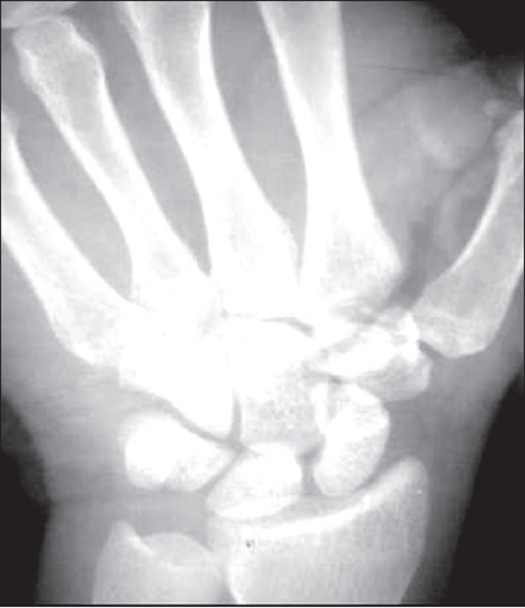
X-rays of wrist (A.P. view) showing fracture of trapezoid

**Figure 2:**
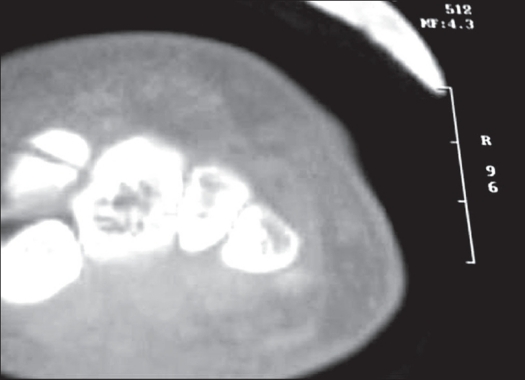
Computer tomography scan shows fractures of trapezoid and anterior cortical break in capitate bone

Radiological examination showed a displaced oblique fracture of the body of trapezoid [Figure 1]. The computerized tomography (CT) showed a fracture of trapezoid and break in anterior cortex of capitate [Figure 2].

**Figure 3:**
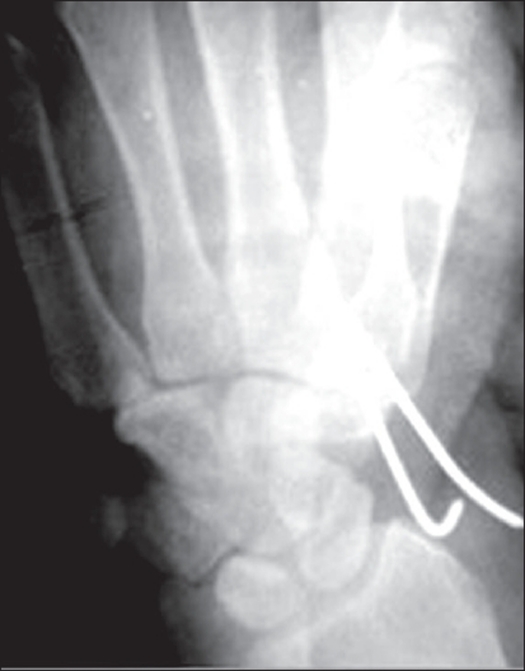
Postoperative X-ray of the wrist (A.P.) showing trapezoid fracture stabilized wirh Kirschner wire

On the day of injury, after primary wound care, open reduction and internal fixation of trapezoid with Kirschner wires was carried out [Figure 3]. All tendons, capsule, retinaculum and adductor pollicis muscle were promptly repaired. Followup CT scan showed a stable fixation of trapezoid. A dorsal plaster of Paris slab with the wrist in extension and slight flexion at carpometacarpal joint was applied for six weeks postoperatively. Six weeks after the operation the Kirschner wires were removed. Active physiotherapy was started at 6 weeks.

## DISCUSSION

The trapezoid forms a very stable, relatively immobile joint with the second metacarpal base distally. It is bound by strong ligaments to the trapezium radially, capitate ulnarly and scaphoid proximally. The trapezoid is shaped like a keystone and is widest dorsally. It is the least commonly injured carpal bone, involved in less than one per cent of carpal injuries.^1^,^2^ There are six reports of the trapezoid fracture in the English literature.^5^,^11^ However, isolated fracture of the trapezoid is extremely rare and only two cases have been previously reported.^5^,^11^ The fracture of the trapezoid has not been reported with any other carpal fractures.^9^ An axial force applied to the second metacarpal usually produces fractures of the trapezoid.^2^–^4^ In the present case, fracture appeared to be caused by the impact of a sharp object. Computerized tomography was useful in attaining a definitive diagnosis and evaluation of fractures of the trapezoid.^6^ Since little has been written on the treatment of trapezoid fractures, a standard treatment method for trapezoid fractures has not been established. In two cases conservative therapy were chosen^5^,^6^ and one case required removal of a small fragment of trapezoid.^7^ On the other hand, fractures of the trapezoid sometimes require open reduction and internal fixation.^2^,^3^,^8^,^11^ In the present case, as displacement was noted and the fracture being open, open reduction and internal fixation was performed.[11]
